# Ischemic preconditioning in liver resection surgery: insights and future directions

**DOI:** 10.1097/JS9.0000000000001040

**Published:** 2024-01-08

**Authors:** Kuo-Chuan Hung, I-Wen Chen, Ping-Hsin Liu

**Affiliations:** a Department of Anesthesiology, Chi Mei Medical Center; b Department of Anesthesiology, Chi Mei Medical Center, Liouying, Tainan City; c Department of Anesthesiology, E-Da Dachang Hospital, I-Shou University, Kaohsiung City, Taiwan

*Dear Editor,*

We commend the recent work of de Oliveira *et al*.^1^ for their rigorous and detailed systematic review of 17 randomized clinical trials, focusing on the impact of ischemic preconditioning (IPC) in enhancing surgical outcomes in liver resections. Their meticulous approach and robust methodology provided valuable insights into this complex area of surgical practice^1^. Given the inherent complexities and significant risks associated with liver resection procedures, any strategy, such as IPC, that offers potential benefits in reducing complications and promoting patient recovery is of paramount interest within the medical community. The study’s findings that IPC does not extend surgical duration, yet notably diminishes intraoperative bleeding (Mean Difference: −49.97 ml; 95% CI: −86.32), reduces the requirement for blood transfusions [relative risk (RR): 0.71; 95% CI: 0.53–0.96], and reduces the risk of postoperative ascites (RR: 0.40; 95% CI: 0.17–0.93) are particularly striking^1^. These outcomes suggest that IPC could be a significant asset in liver resection surgeries, potentially leading to improved patient outcomes and more efficient utilization of healthcare resources.

Several studies have indicated that excessive blood transfusion can adversely affect patient outcomes^2,3^. Thus, the ability of IPC to reduce the need for blood transfusion is a crucial finding in the meta-analysis^1^. Nevertheless, it is important to note that the current evidence supporting the routine application of IPC in surgical procedures may be insufficient. A cautious approach toward implementing any new surgical technique or intervention is imperative until thorough validation is achieved. To examine the robustness of evidence, we performed trial sequential analysis, a method used to evaluate the cumulative evidence in meta-analyses, using raw data from the original meta-analyses^1^. This approach helps in determining the reliability and sufficiency of the data. TSA was performed with TSA viewer version 0.9.5.10 Beta (www.ctu.dk/tsa) as previously reported^4,5^. As depicted in Figure 1, while the *Z*-curve surpassed the conventional statistical significance boundary, it did not cross the trial sequential monitoring boundary, indicating that the current evidence is insufficient to conclusively establish the benefits of IPC in reducing the need for blood transfusion. Nonetheless, the previous meta-analysis^1^ does a commendable job of elucidating the association between IPC and the need for blood transfusion, thereby paving the way for future research. Our additional analysis indicates the necessity for additional research before reaching definitive conclusions on the effectiveness of IPC and recommending alterations to clinical practice.

**Figure 1:**
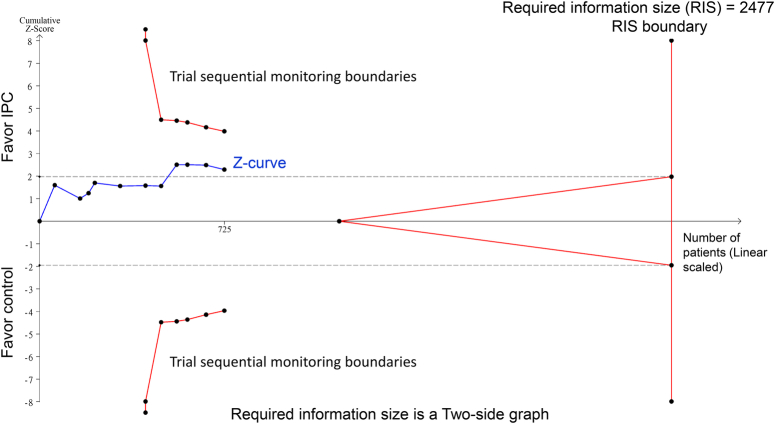
Trial sequential analysis evaluating the efficacy of ischemic preconditioning (IPC) in reducing the need for blood transfusion during liver resection surgery. The *Z*-curve surpasses the conventional statistical significance boundary (gray line) but does not cross the trial sequential monitoring boundary, indicating insufficient evidence to conclusively determine the efficacy of IPC in reducing the need for perioperative blood transfusions. More trials are needed to reach firm conclusions.

## Ethical approval

Not applicable.

## Consent

Not applicable.

## Sources of funding

No external funding was received for this study.

## Author contribution

K.-C.H. and P.-H.L.: wrote the main manuscript text; I-W.C.: prepared Figure 1. All authors read and approved the final version of the manuscript.

## Conflicts of interest disclosure

The authors declare no conflicts of interest.

## Research registration unique identifying number (UIN)

Not applicable.

## Guarantor

Kuo-Chuan Hung.

## Data availability statement

The datasets used and/or analyzed in the current study are available from the corresponding author upon reasonable request.

## Provenance and peer review

This paper was not invited.
